# Editorial: New challenges and future perspectives in cellular neuroscience

**DOI:** 10.3389/fncel.2023.1136888

**Published:** 2023-01-25

**Authors:** Natalia Arias, Gabriella Panuccio, Markus Rothermel

**Affiliations:** ^1^Institute of Neurosciences of the Principality of Asturias (INEUROPA), Oviedo, Spain; ^2^Health Research Institute of the Principality of Asturias (Instituto de Investigación Universitaria del Principado de Asturias), Oviedo, Spain; ^3^Department of Psychology, Faculty of Life and Natural Sciences, Brain and Behavior Group, Nebrija University, Madrid, Spain; ^4^Enhanced Regenerative Medicine, Istituto Italiano di Tecnologia, Genova, Italy; ^5^Institute for Physiology and Cell Biology, University of Veterinary Medicine, Foundation, Hannover, Germany

**Keywords:** therapies, nervous system, techniques, health and disease, translational research

This Research Topic highlights recent advancements in our understanding of basic processes in cellular neuroscience with a special focus on pathological mechanisms that could lead to the development of promising new therapeutic strategies in the future. Over the last decade, there has been growing interest in translational neuroscience research, advancing our understanding of the mechanisms underlying cell function in the nervous system in health and disease, partially spurred by well-funded research consortia. Undoubtedly, translational research is one of the most pressing matters in neuroscience. However, so far, big promised breakthroughs like curing Alzheimer's or understanding superior cognitive processes are not only lacking but seem to be even more distant than ever. Likely due to these unrealistic promises of the past of what neuroscience would be able to achieve by now, other more incremental but nonetheless fundamental advances have yet to reach broader public awareness. Partially, these advances have been enabled by improvements in research techniques like somatic cell reprogramming or single-cell RNA-sequencing that have gradually become more accessible to the research community worldwide. By using these techniques and employing them on very different research questions, new experiments have been performed and amounts of data collected that were hard to imagine only a couple of years ago. This current state of the art will define the (technical) progress that needs to be made in the next decade.

This Research Topic comprises a balanced mix of original research and review articles that contribute to current views as well as challenges and future perspectives in different fields of the neurosciences, such as the study of peripheral nervous system inflammation through transcriptomic analysis and molecular mechanisms of blood-brain barrier dysfunction. Brain development and function have been approached in relation to the role of the environment and the contribution of the gut-microbiota-brain axis. The potential of transcranial stimulation to improve brain plasticity has been examined and a novel model for controlling synaptic transmission proposed. Finally, somatic cell reprogramming has been examined for treating spinal cord injury. This topic sheds light on the techniques and current knowledge in the field that might soon lead to new therapies to understand and treat brain dysfunctions.

The study by Liu et al. investigates inflammatory transcriptional alterations of mouse trigeminal ganglion neurons. By using single-cell RNA sequencing in the peripheral system of mice this original research paper revealed the heterogeneity of trigeminal neurons and their diverse neuronal transcriptomic responses to orofacial inflammation. Orofacial pain is a debilitating pain condition and a serious public health problem (Banigo et al., 2018). The observed heterogeneity in trigeminal ganglion neurons is surprising given that classically besides of their area of innervation, these neurons were considered relatively homogenous. The results of the present study might provide new ideas for the development of therapeutic strategies for orofacial inflammatory pain.

The review by Chen et al. shed light on the molecular mechanisms of ferroptosis and its role in blood-brain barrier dysfunction. Ferroptosis, which in recent years has attracted more and more attention due to its possible role in nervous system disorders, is a cell death mode caused by iron-mediated lipid peroxidation accumulation (Li et al., 2020). The blood-brain barrier protects the brain from dangerous chemicals in the bloodstream thereby maintaining a tightly regulated microenvironment for neuronal signaling. Recently it has been shown that lipid peroxidation and iron accumulation are related to barrier dysfunction. This review examines the relationship between ferroptosis and blood-brain barrier dysfunction which may reveal new targets for the treatment of brain diseases.

The importance of epigenetics, the regulation of gene expression by environmental factors independently of DNA sequences, has constantly increased in recent years. The article by Vázquez-Ágredos et al. provides a systemic review of epigenetic regulation of the environmental impact on adolescent neurobehavioral development. Adolescent behavior is not only characterized by novelty-seeking but also by increased stress responsivity (Spear, 2013) and early life stress has been linked to a higher risk for alcohol and drug use disorders (Kirsch and Lippard, 2022). This review focuses on the miRNA expression patterns in the rodent brain with a special interest in the impact of stress and drugs such as amphetamine, cocaine, nicotine, cannabis, and ketamine. The authors point to a dynamic epigenetic network sensitive to environmental events with distinctive changes across adolescence. A deeper understanding of environmental threats during this critical period in neurodevelopment is fundamental for the understanding of psychiatric and addictive disorders emerging at this stage.

Other external factors such as diets or alcohol consumption can impact brain function *via* the gut-brain axis. The original research article by Higarza et al. proposes an important extension namely the liver-gut-brain axis. They investigated the dynamic changes of gut microbiota in different rodent models along the liver disease spectrum and found that liver damage affected the diversity and bacterial community structure. Moreover, they investigated potential changes in brain metabolism and cognition. They found changes in brain oxidative metabolism in many brain areas including the prefrontal, retrosplenial, and perirhinal cortices, as well as the amygdala and mammillary bodies, potentially accounting for the observed impact on working memory. In the future, gut microbiota could be more specifically targeted to design more effective treatments, for specific stages of liver disease.

Improving neuronal plasticity can be a new therapeutic avenue for several brain disorders linked to impaired learning and memory. In this regard, the mini-review “Tuning brain networks: The emerging role of transcranial direct current stimulation on structural plasticity” (Barbati et al.) discusses transcranial direct current stimulation effects on structural plasticity, neuronal rewiring and the underlying molecular changes. Transcranial direct current stimulation is a non-invasive brain stimulation technique that is used to treat a range of neurological disorders ranging from stroke and epilepsy to movement disorders, Parkinson's disease, and Alzheimer's disease (Floel, 2014). So, a deeper understanding of transcranial direct current stimulation effects is a prerequisite for treating diverse brain dysfunctions.

In search for mechanisms of synaptic plasticity, the article by Barnett and Goult proposes a new concept of how cells compute and store information using intracellular scaffolding proteins (The MeshCODE). The theory proposes that the cells' entire cytoskeletal architecture serves to perform mechanical computation. In their original research paper, they report a Gearbox-like mechanism for the dynamic regulation of synaptic function. Based on biophysical rules and experimentally derived distances, the authors provide a novel perspective on biological information and the next years will tell if more evidence for their theory can be obtained.

The article by Yang et al. provides a review of the transcription factors, genes and microRNAs that can mediate somatic cell reprogramming to repair the injured spinal cord. Despite tremendous scientific efforts, spinal cord injury is still challenging to treat. Early discoveries were promising (Schwab, 2004), however, it became evident that axonal regeneration is just one part of the problem. Importantly necrosis/apoptosis at the injured area leading to glia cell dysfunction might even be the bigger hurdle to overcome. Somatic cell reprogramming is a promising technology that has gradually found its way into many different neuroscience areas and since it can be used to reprogram glia and neural progenitor cells into neurons or oligodendrocytes, it represents a promising technique for promoting spinal cord repair. Somatic cell reprogramming provides new possibilities for achieving functional recovery after spinal cord injury that could soon lead to new treatments.

Altogether, this Research Topic highlights data from different model organisms, neuronal as well as glial cells and incorporates genetic, physiological, molecular and computational approaches to offer new perspectives in many different areas of cellular neuroscience. Whilst the community should be proud of their advances, there is still a long way to go to resolve many major neuroscientific issues. We hope that this Research Topic will be inspirational to those who are willing to push beyond the boundaries of current paradigms and thinking in neuroscience, aiming at furthering our understanding of the nervous system and ultimately bringing novel, effective therapies for disorders of the nervous system, which carry today the highest global burden of disease.

## Author contributions

MR wrote the first draft of the manuscript. All authors contributed to manuscript revision, read, and approved the submitted version.
